# Cellular Proteomes Drive Tissue-Specific Regulation of the Heat Shock Response

**DOI:** 10.1534/g3.116.038232

**Published:** 2017-01-30

**Authors:** Jian Ma, Christopher E. Grant, Rosemary N. Plagens, Lindsey N. Barrett, Karen S. Kim Guisbert, Eric Guisbert

**Affiliations:** Department of Biological Sciences, Florida Institute of Technology, Melbourne, Florida 32901

**Keywords:** stress response, protein folding, proteostasis, HSF1, heat shock response

## Abstract

The heat shock response (HSR) is a cellular stress response that senses protein misfolding and restores protein folding homeostasis, or proteostasis. We previously identified an HSR regulatory network in *Caenorhabditis elegans* consisting of highly conserved genes that have important cellular roles in maintaining proteostasis. Unexpectedly, the effects of these genes on the HSR are distinctly tissue-specific. Here, we explore this apparent discrepancy and find that muscle-specific regulation of the HSR by the TRiC/CCT chaperonin is not driven by an enrichment of TRiC/CCT in muscle, but rather by the levels of one of its most abundant substrates, actin. Knockdown of actin subunits reduces induction of the HSR in muscle upon TRiC/CCT knockdown; conversely, overexpression of an actin subunit sensitizes the intestine so that it induces the HSR upon TRiC/CCT knockdown. Similarly, intestine-specific HSR regulation by the signal recognition particle (SRP), a component of the secretory pathway, is driven by the vitellogenins, some of the most abundant secretory proteins. Together, these data indicate that the specific protein folding requirements from the unique cellular proteomes sensitizes each tissue to disruption of distinct subsets of the proteostasis network. These findings are relevant for tissue-specific, HSR-associated human diseases such as cancer and neurodegenerative diseases. Additionally, we characterize organismal phenotypes of actin overexpression including a shortened lifespan, supporting a recent hypothesis that maintenance of the actin cytoskeleton is an important factor for longevity.

The HSR was first identified >50 yr ago as a cellular, transcriptional response to increased temperature (Ritossa 1962). This response has subsequently been shown to be induced by protein misfolding, resulting in activation of the heat shock factor 1 (HSF1) transcription factor [reviewed in Guisbert and Morimoto (2013) and Guisbert *et al.* (2008)]. More recently, the HSR has been shown to have an important role in a variety of human diseases. In diseases of protein misfolding, such as Alzheimer’s disease, animal models have shown that activation of the HSR is beneficial [reviewed in Hipp *et al.* (2014)]. On the other hand, the HSR is constitutively active in several cancers, and inhibition of the HSR appears to be beneficial in cancer models [reviewed in Dai and Sampson (2016)]. While it remains to be seen whether manipulation of the HSR will prove to be a viable route for new disease therapeutics, a more complete understanding of HSR regulation will contribute to both basic biology and insight into human disease.

HSF1 senses protein folding through the HSP70 and HSP90 molecular chaperones, which bind to newly synthesized or misfolded proteins [reviewed in Hartl *et al.* (2011)]. Both HSP70 and HSP90 bind directly to HSF1 and inhibit its activity when the level of misfolded proteins is low (Shi *et al.* 1998, Zou *et al.* 1998). When misfolded proteins accumulate, chaperones are titrated away from HSF1, therefore liberating HSF1 to upregulate a set of genes called heat shock genes. Heat shock genes include many of the molecular chaperones themselves, thus forming a negative feedback loop that elegantly links the total amount of chaperones in the cell to the cellular need for chaperones.

This molecular model for HSR regulation predicts a cell-autonomous response that couples chaperone expression to protein misfolding within each cell. Moreover, the central regulatory module of the HSR, consisting of HSF1, HSP70, and HSP90, is thought to be broadly expressed. Therefore, stress-sensing mechanisms were assumed to be conserved across all tissues in a multicellular organism. However, in a recent screen for HSR regulators, we discovered that regulators of the HSR display exquisite tissue specificity (Guisbert *et al.* 2013). We identified 52 negative regulators of the HSR, including HSP70 and HSP90. Unexpectedly, we found that each of the 50 new HSR regulators induced the HSR in only a subset of tissue types that were induced by HSP70 and HSP90.

One set of new regulators that we identified contains the eight subunits of the TRiC/CCT chaperonin complex. Chaperonins are a class of ATP-dependent molecular chaperones that form large, barrel-shaped complexes that encapsulate protein substrates [reviewed in Lopez *et al.* (2015)]. The best characterized chaperonin is GroEL/GroES in prokaryotes. TRiC/CCT is the cytoplasmic chaperonin in eukaryotic cells that contains two hetero-oligomeric rings with eight subunits each. TRiC/CCT is essential for the proper folding of actin and tubulin and is estimated to participate in the folding of 10% of the proteome. We found that knockdown of each of the eight TRiC/CCT subunits causes induction of the HSR in muscle tissue, but not in the intestine or reproductive tissue. Importantly, regulation of the HSR by TRiC/CCT has been shown to be conserved in cultured human cell lines and involves direct regulation of HSF1 (Neef *et al.* 2014).

Another class of new HSR regulators includes subunits of the SRP and other components of the secretory pathway. The SRP is a well-conserved complex that contains both protein subunits and noncoding RNAs, which recognize signal sequences in nascent proteins, and targets them to the endoplasmic reticulum [reviewed in Elvekrog and Walter (2015)]. Knockdown of SRP subunits induces the HSR in the intestine but not in the muscle. Similar to TRiC/CCT, regulation of the HSR by SRP appears to be conserved across multiple species as components of the secretory pathway have been shown to directly regulate the HSR in *Escherichia coli* (Lim *et al.* 2013).

The tissue-specific regulation of the HSR highlights an import gap in our basic understanding of HSR regulation, which comes largely from unicellular organisms and cultured cells. Here, we investigate the molecular basis for tissue-specific HSR regulation in a multicellular organism for two distinct subsets of HSR regulators and show that the unique protein folding requirements of each tissue help to drive distinct patterns of HSR induction.

## Materials and Methods

### Nematodes

*Caenorhabditis elegans* were maintained at 20° using standard procedures. Worms were synchronized by bleaching with hypochlorite and hatching overnight in M9 buffer. All nematode strains were derived from the N2 Bristol wild-type strain. The following strains were used: (1) AM446 rmIs223[*C12C8.1p*::*gfp*;*rol-6(su1006)*] (Morley and Morimoto 2004); (2) EAG001 *rmIs223*[*C12C8.1p*::*gfp*;*rol-6(su1006)*], *eagEx1*[*let-858p*::*act-4*;*myo-2p*::*rfp*]; and (3) EAG003 rmIs223[*C12C8.1p*::*gfp*;*rol-6(su1006)*], *eagIs1*[*let-858p*::*act-4*;*myo-2p*::*rfp*]. Plasmid pEAG58 was constructed using the Gateway system to combine an *act-4* ORF (Reboul *et al.* 2003) with the *let-858* promoter and the *unc-54* 3′−UTR. Strain EAG001 was generated by microinjection of plasmid pEAG58 along with a *myo-2p*::*rfp* fluorescent marker into strain AM446. Strain EAG003 was generated by irradiation of strain EAG001, selection for integration, and backcrossing 4 times. The p-values represent raw p-values.

### RNAi

RNAi was induced using a bacterial feeding approach with the Ahringer RNAi library (Kamath *et al.* 2003). Bacterial cultures were grown overnight in LB and induced with 1 mM IPTG for at least 2 hr in liquid cultures and on plates. RNAi was initiated at the L2/L3 stage of development by synchronizing worms at the L1 larval stage and then culturing them for 19 hr on OP50 plates. Nematodes were then grown to adulthood on RNAi plates ∼48 hr prior to analysis.

### Imaging

Worms were mounted on 3% agarose pads on a glass slide, immobilized in a drop of 1 mM levamisole, and imaged using a Nikon C1Si multi-spectral laser scanning confocal microscope and Nikon EZ-C1 software. Each individual worm was visually scored for induction of the fluorescent reporter. Unhatched eggs were collected in M9 after bleaching and directly visualized using a Zeiss Axioskop2 brightfield microscope and analyzed using Jenoptik’s ProgRes Capture Pro software.

### qRT-PCR

RNA was isolated from whole animals using TRIzol (Invitrogen) according to standard protocols. DNA was removed using a DNA-free DNA Removal Kit (Ambion), cDNA synthesis was performed using an iScript cDNA Synthesis Kit (Bio-Rad), and qPCR was performed using iQ SYBR Green Supermix (Bio-Rad) using a CFX Connect Real Time System (Bio-Rad). 18S RNA was used as a normalization control.

### Phenotypic assays

Thrashing assays were conducted on day 1 of adulthood. Individual worms were picked into a drop of M9 buffer on a glass slide, acclimated for 1 min, and then the number of thrashing movements were visually counted in a 30 sec period using a dissecting microscope. Egg laying assays were conducted by counting the number of eggs laid in each 24 hr period from larva to cessation of egg laying. Total brood size was then calculated for each worm. Egg hatching was assayed by transferring young adult worms to a new plate, allowing egg laying to occur for 3 hr, and then removing the adult worms. The eggs were then incubated for 24 hr and the number of hatched larvae were counted. Development was assayed over time after synchronization with visual inspection of gonad morphology.

### Lifespan

Lifespan experiments were performed at 20° in the absence of FUDR. Worms were transferred to new plates at least every other day to separate adults from progeny. Lifespan data were analyzed using a log-rank test in OASIS (Online Application for the Survival Analysis of Lifespan Assays Performed in Aging Research).

### Data availability

All strains and reagents are available upon request. The authors state that all data necessary for confirming the conclusions presented in the article are represented fully within the article.

## Results

### Expression of TRiC/CCT

**Figure 1:**
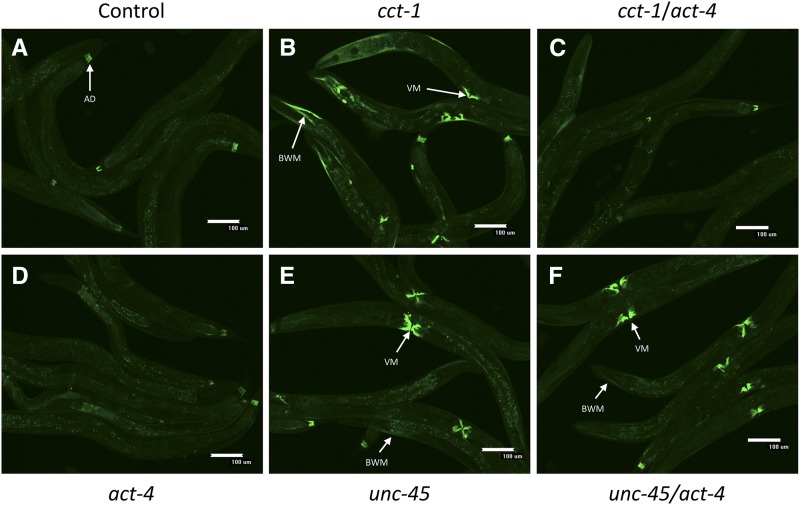
Muscle-specific induction of the HSR by *cct-1* knockdown is dependent on *act-4*. Fluorescent images are shown of strain AM446 containing a GFP-based HSR reporter. (A) Control, nonsilencing RNAi worms only display autofluorescence and constitutive reporter expression in the AD. (B) Knockdown of *cct-1* induces the HSR reporter in muscle tissue as indicated with the arrows labeled VM and BWM. (C) Knockdown of *act-4* prevents muscle-specific induction by *cct-1* knockdown. (D) Knockdown of *act-4* alone does not induce the HSR. (E) Knockdown of *unc-45* also induces the HSR in muscle tissue. (F) Knockdown of *act-4* does not affect HSR induction by *unc-45*. In these experiments, the single gene RNAi knockdowns were diluted with control, nonsilencing RNAi so that they had the same dosage as the double RNAi knockdowns. Quantitation of the results is given in Table 1. AD, anal depressor muscle; BWM, body wall muscle; GFP, green fluorescent protein; HSR, heat shock response; RNAi, RNA interference; VM, vulva muscle.

**Figure 2:**
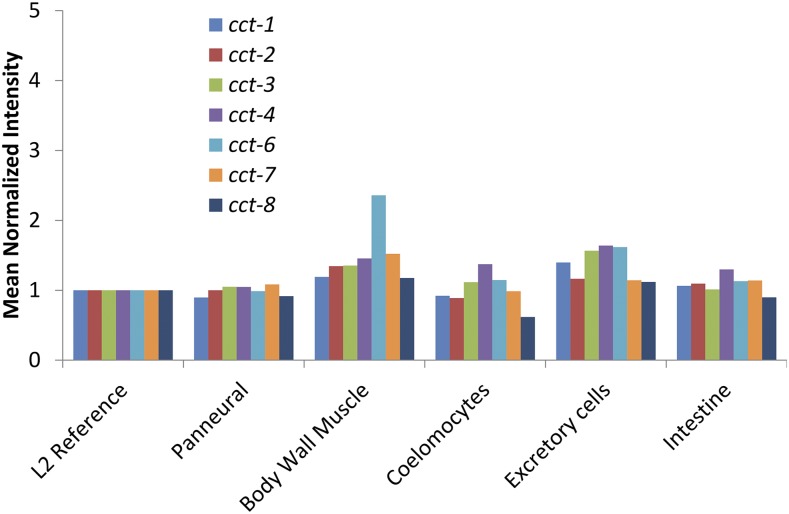
TRiC/CCT subunit expression is not strongly enriched in muscle tissue. Mean normalized signal intensity for gene expression data are shown for each of the available TRiC/CCT subunits in various tissues from L2-staged larval worms relative to the L2 whole worm reference control. Data were taken from the modENCODE project (Spencer *et al.* 2011).

The HSR is regulated by highly conserved genes with well-established roles in cellular protein folding, yet these genes regulate the HSR primarily in a tissue-specific manner (Guisbert *et al.* 2013). To uncover mechanisms that drive tissue specificity, we first analyzed tissue-specific HSR regulation by the TRiC/CCT chaperonin complex. Knockdown of *cct-1*, a subunit of the complex, induces the HSR in muscle tissue, but not in the intestine or reproductive tissues (Figure 1 and Figure S1 in Supplemental Material, File S1). As reported, this induction is quite distinct from induction by heat shock (Figure S2 in File S1). The simplest explanation for this muscle-specific induction might be that *cct-1* and other TRiC/CCT subunits are only expressed in muscle tissue. However, this possibility is unlikely given that TRiC/CCT is required for the folding of actin and tubulin, which are essential components of the cytoskeleton. Supporting this, a functional role for the TRiC/CCT chaperonin in actin and tubulin folding in the *C. elegans* intestine has been characterized (Saegusa *et al.* 2014). Additionally, previous qualitative analyses of expression patterns of TRiC/CCT subunits have suggested a ubiquitous pattern of expression across tissues (Lundin *et al.* 2008). However, some fluorescent reporter constructs suggest that TRiC/CCT is enriched in muscle tissue (Leroux and Candido 1997). To resolve this discrepancy, we queried the expression of TRiC/CCT subunits from a genomic analysis of gene expression patterns in *C. elegans* as part of the modENCODE consortium (Spencer *et al.* 2011). These data were generated using tagged RNA-binding proteins expressed in a tissue-specific manner to examine the tissue-specific expression of endogenous mRNAs. In this dataset, the expression of TRiC/CCT subunits is relatively uniform across different cell types (Figure 2). Similarly, expression of the central regulatory module of the HSR, including HSP70, HSP90, and HSF1, also displays little or no tissue specificity (Figure S3 in File S1). This indicates that the muscle-specific effect of TRiC/CCT on the HSR cannot be explained by the expression pattern of TRiC/CCT or other major regulators of the HSR.

### Requirement for actin in TRiC/CCT muscle-specific HSR regulation

If HSF1 and TRiC/CCT are both expressed across many tissues, then what could explain their specific genetic interaction in muscle tissue? For the well-established regulation of HSF1 by the HSP70 and HSP90 molecular chaperones, HSR regulation is mediated by the ratio between the chaperones and their substrates (Craig and Gross 1991). Therefore, we considered whether the substrates of TRiC/CCT might influence its tissue specificity. Actin is one of the major substrates of TRiC/CCT, (Lopez *et al.* 2015), therefore, we tested whether actin expression contributed to TRiC/CCT-mediated regulation of the HSR.

There are five actin isoforms in *C. elegans*, and four of these (*act-1*, *act-2*, *act-3*, and *act-4*) are 99% identical. The expression patterns of actin are not well-established as reporter fusions suggest that the four similar isoforms are enriched in muscle tissues, whereas *in situ* analysis reveals broader expression patterns (Stone and Shaw 1993, Macqueen *et al.* 2005). The remaining actin isoform, *act-5*, is 93% identical and expressed in the intestine where it has important roles in the formation of intestinal microvilli. Analysis of actin isoform expression patterns from the modENCODE dataset reveals that there is not a robust enrichment of actin expression in muscle tissue relative to the whole worm reference (Figure S2 in File S1). Nevertheless, there is enrichment for *act-5* and a decrease in *act-1*, *act-2*, and *act-4* expression in the intestine, resulting in an increased ratio of muscle to intestine expression. Supporting this, dominant mutants in actin isoforms preferentially affect muscle tissue, indicating a functional enrichment (Waterston *et al.* 1984).

**Figure 3:**
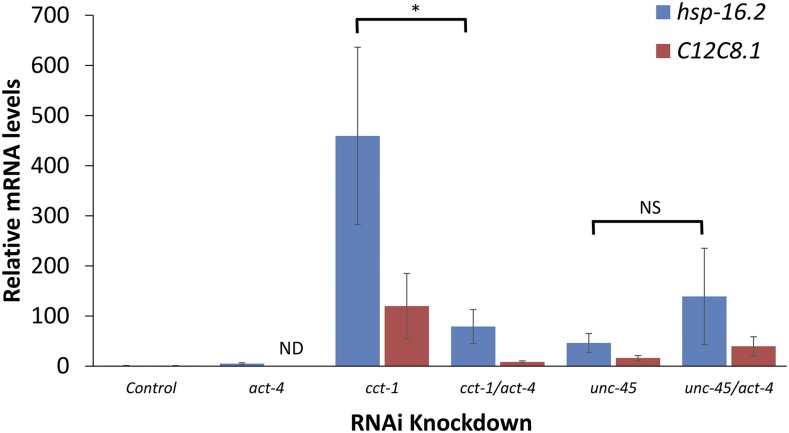
HSR induction of endogenous HSR genes by *cct-1* knockdown is dependent on *act-4*. qRT-PCR analysis of two endogenous HSR genes, *hsp-16.2* and *C12C8.1* (*hsp-70*), reveals that HSR induction by *cct-1* knockdown is decreased by knockdown of *act-4*. Both genes showed the same trend but the effect was only significant for *hsp-16.2* due to the increased variability of *C12C8.1* (p-value = 0.05 for *hsp-16*.2 and p-value = 0.09 for *C12C8.1*). In contrast, HSR induction by *unc-45* knockdown was not decreased by knockdown of *act-4* and is therefore marked as NS (*n* ≥ 6 for all samples except for *C12C8.1* in the act-4 knockdown control where the signal was below the detection limit for many samples and is therefore marked as ND). HSR, heat shock response; mRNA, messenger RNA; ND, not determined; NS, not significant; qRT-PCR, quantitative real time polymerase chain reaction; RNAi, RNA interference.

We next tested whether the enrichment of actin in the muscle could influence the muscle-specific HSR induction from TRiC/CCT knockdown. We found that knockdown of the actin isoform *act-4* resulted in inhibition of HSR induction caused by *cct-1* knockdown (Figure 1). In contrast, *act-4* knockdown did not affect HSR induction in the muscle by knockdown of *unc-45*, an HSR regulator unrelated to TRiC/CCT. Additionally, induction of the reporter by heat shock was not reduced upon *act-4* knockdown (Figure S2 in File S1). Similar results were obtained by measuring induction of endogenous HSR genes using qPCR (Figure 3). Knocking down the three other broadly expressing actin isoforms also affects HSR induction by *cct-1*, indicating that this effect is not specific to any one actin isoform (Table 1). However, even though the RNAi constructs were designed to target specific actin isoforms, it has been previously shown that these constructs can knockdown multiple isoforms due to the high degree of sequence conservation among the isoforms (Velarde *et al.* 2007). Nevertheless, because the actin isoforms are thought to be largely redundant, this lack of specificity does not impact the conclusions.

**Table 1:** Dependence of muscle-specific HSR induction on actin isoforms

Induction (%)	Control (%)	*act-1* (%)	*act-2* (%)	*act-3* (%)	*act-4* (%)
*cct-1*	94 ± 3	10 ± 6	28 ± 5	25 ± 5	53 ± 3
*unc-45*	92 ± 4	94 ± 6	90 ± 4	83 ± 3	100 ± 0

Quantification of the percentage of worms with heat shock response (HSR) reporter induction from strain AM446 containing a green fluorescent protein (GFP)-based HSR reporter after double RNA interference (RNAi) knockdown of *cct-1* or *unc-45* with control, nonsilencing RNAi, *act-1*, *act-2*, *act-3*, and *act-4* actin isoform knockdowns. Actin isoform knockdown leads to a decrease in the percentage of worms with HSR induction upon *cct-1* knockdown (*act-1* p-value = 2.8E−07, *act-2* p-value = 2.8E−07, *act-3* p-value = 2.4E−07, and *act-4* p-value = 2.4E−05), but does not affect the percentage of worms with HSR induction upon *unc-45* knockdown (*act-1* p-value = 0.45, *act-2* p-value = 0.33, *act-3* p-value = 0.1, and *act-4* p-value = 0.16). Data are from at least three trials and error represents SEM.

### Sufficiency of actin for TRiC/CCT tissue-specific HSR regulation

**Figure 4:**
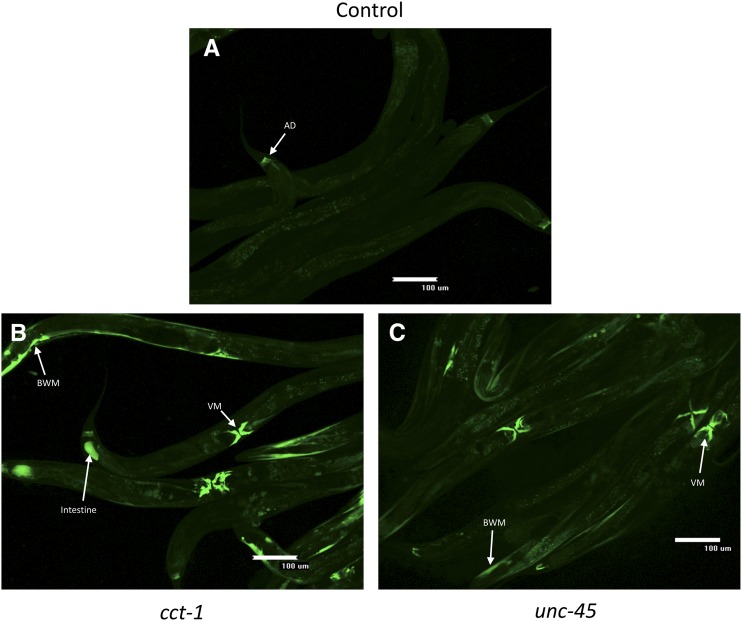
Actin overexpression expands the tissue-specific pattern of HSR induction by *cct-1* knockdown. Fluorescent images are shown of strain EAG001, which overexpresses the actin isoform *act-4* and contains a GFP-based HSR reporter after RNAi with nonsilencing control, *cct-1*, or *unc-45*. (A) Control, nonsilencing RNAi worms only display autofluorescence and constitutive reporter expression in the AD. (B) Knockdown of *cct-1* results in HSR induction in the intestine in addition to VM and BWM. Intestinal HSR induction occurs in 30 ± 9% of worms (*N* = 4 trials, error is SEM). (C) Knockdown of *unc-45* results in HSR induction in muscle tissue, but induction was not observed in the intestine. AD, anal depressor muscle; BWM, body wall muscle; GFP, green fluorescent protein; HSR, heat shock response; RNAi, RNA interference; VM, vulva muscle.

Having established that the levels of actin are necessary for HSR induction by TRiC/CCT knockdown, we next asked whether actin was sufficient to drive induction of the HSR by TRiC/CCT in other tissues. Therefore, we generated a transgenic line containing the *act-4* actin isoform expressed under the control of a broad, *let-858* promoter. We found that overexpression of *act-4* is not sufficient to induce the HSR on its own (Figure 4A). However, overexpression of *act-4* combined with knockdown of *cct-1* led to HSR induction in the intestine (Figure 4B). In contrast, *act-4* overexpression did not sensitize the intestine to HSR induction by *unc-45* knockdown (Figure 4C). These results indicate that actin expression is sufficient to sensitize the intestine to induce the HSR upon knockdown of TRiC/CCT. Therefore, HSR regulation by TRiC/CCT is not solely mediated by the levels of TRiC/CCT, but is instead determined by the ratio of TRiC/CCT to its substrates.

### Organismal phenotypes for actin

**Figure 5:**
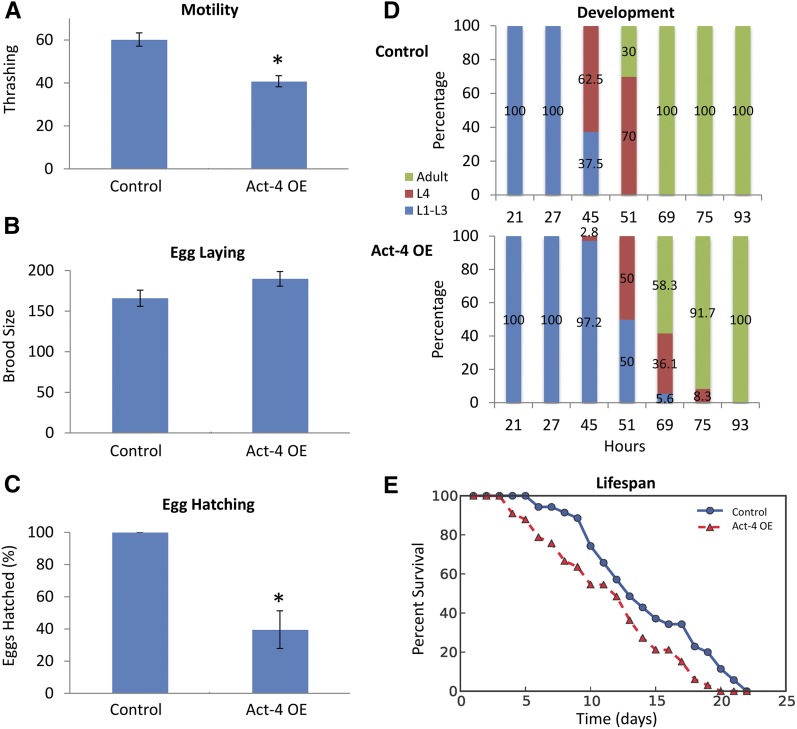
Phenotypic effects of Act-4 OE. Various phenotypes were measured in strain EAG003, which contains an integrated Act-4 OE construct with an HSR reporter, and in the control strain AM446, which only contains the HSR reporter. All error bars represent SEM. (A) Actin OE causes a decrease in motility represented by a 32% decrease in thrashing rate per 30 sec (p-value = 0.00001, *n* = 30 worms). (B) Actin OE does not cause a decrease in brood size (*n* ≥ 35 worms). (C) Actin OE causes a decrease in hatching rate measured 24 hr after laying (p-value = 0.01, *n* ≥ 144 eggs). (D) Actin OE delays but does not impair postembryonic development (*n* ≥ 69 worms). (E) Actin OE causes a decrease in lifespan (p-value = 0.02, *n* ≥ 33 worms). Lifespan data were analyzed using a log-rank test in OASIS (Online Application for the Survival Analysis of Lifespan Assays Performed in Aging Research). HSR, heat shock response; OE, overexpression.

We next characterized other phenotypes that arise from actin overexpression. We generated an integrated transgenic worm strain overexpressing *act-4* by irradiating the extrachromosomal *act-4* overexpression line and selecting for integration. The integrated line was validated to be similar to the extrachromosomal line with respect to induction of the HSR in the intestine upon *cct-1* knockdown (Figure S4 in File S1). Next, expression levels were quantitated using qPCR, and *act-4* was found to be ∼2.6-fold overexpressed relative to wild-type worms (Figure S5 in File S1). This level of overexpression is mild since it represents just one of the five actin isoforms. Given the extensive post-translational regulation of actin localization and polymerization, it is unclear whether mild overexpression would cause serious functional consequences. However, as actin is one of the most abundant proteins in the cell, even mild overexpression could cause significant perturbations. To investigate muscle function, motility was measured using a thrashing assay and a 30% decrease in motility was observed (Figure 5A). Given the important role of actin in muscle tissue, this decrease is consistent with a mild perturbation of cellular function. Next, the role of actin in early development was investigated by measuring the brood size and the egg hatching rate (Figure 5, B and C). No decrease was observed in the number of eggs laid, but consistent with the established roles of actin in early development, less than half of the eggs laid were viable. Microscopic analysis of the eggs indicated a defect in gastrulation as unhatched eggs were observed that were arrested before and during the various embryonic stages associated with gastrulation, including the comma and the bean (Figure S5 in File S1). Of the eggs that hatched, they all reached adulthood even though there was a slight developmental delay (Figure 5D). Finally, the lifespan of adult worms was measured to test a recent hypothesis regarding the actin cytoskeleton and aging. Previously, it was shown that disruptions in muscle structure and function are associated with aging (Herndon *et al.* 2002). It was recently shown that overexpression of *pat-10*, a troponin-like protein that regulates the actin cytoskeleton, reverses age-dependent alterations to actin and extends lifespan, suggesting that the actin cytoskeleton has an important role in aging (Baird *et al.* 2014). Supporting this hypothesis, we observed a significant decrease in lifespan upon mild actin overexpression (Figure 5E). This effect was validated by measuring lifespan in the extrachromosomal actin overexpression line where a similar decrease was observed (Figure S6 in File S1).

### Requirement of vitellogenins for intestine-specific HSR regulation by SRP

**Figure 6:**
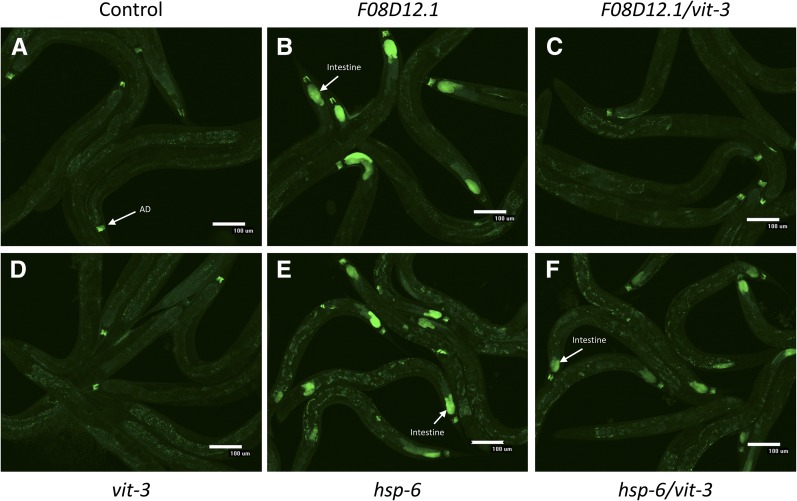
Intestine-specific induction of the HSR by knockdown of the SRP subunit *F08D12.1* (*srpa-72*) is dependent on the secreted vitellogenin *vit-3*. Fluorescent images are shown of strain AM446 containing a GFP-based HSR reporter. (A) Control, nonsilencing RNAi worms only display autofluorescence and constitutive reporter expression in the AD. (B) Knockdown of *F08D12.1* induces the HSR reporter in the intestine. (C) Knockdown of *vit-3* prevents intestine-specific HSR induction by *F08D12.1*. (D) Knockdown of *vit-3* alone does not induce the reporter. (E) Knockdown of *hsp-6* also induces the HSR reporter in the intestine. (F) Knockdown of *vit-3* does not affect reporter induction upon *hsp-6* knockdown. Quantitation of these results is given in Table 2. AD, anal depressor muscle; GFP, green fluorescent protein; HSR, heat shock response; RNAi, RNA interference; SRP, signal recognition particle.

To elucidate whether or not the ratio of HSR regulators to their substrates was a general feature of HSR regulation in *C. elegans*, we next asked whether the same principle applied to HSR regulators in the secretory pathway. Knockdown of the SRP subunit *F08D12.1* causes induction of the HSR in the intestine, but not in muscle tissue (Figure 6). We hypothesized that intestinal cells may be particularly dependent on proper folding in the secretion pathway due to the extremely high expression of the vitellogenin genes. Vitellogenins, or yolk proteins, are synthesized in the intestine, secreted into the pseudocoelomic space, and taken up in eggs through endocytosis (Kimble and Sharrock 1983). We knocked down the two vitellogenins from our RNAi library, *vit-3* and *vit-5*, and found that these genes prevented induction of the HSR upon knockdown of *F08D12.1* (Figure 5 and Table 2). In contrast, they did not affect induction of the HSR in the intestine by knockdown of *hsp-6*, a mitochondrial chaperone. Together, these data indicate that regulation of the HSR by the secretory pathway also involves a balance between the components of the secretory pathway and their substrates, and that this type of regulation is a general feature of the HSR.

**Table 2:** Dependence of intestine-specific HSR induction on vitellogenins

Induction (%)	Control (%)	*vit-3* (%)	*vit-5* (%)
*F08D12.1*	69 ± 10	3 ± 3	0 ± 0
*hsp-6*	47 ± 12	60 ± 15	48 ± 20

Quantification of the percentage of worms with heat shock response (HSR) reporter induction from the *phsp70*::*gfp* reporter strain for double RNA interference (RNAi) knockdown of *F08D12.1* or *hsp-6* with control, nonsilencing RNAi, *vit-3*, and *vit-5* vitellogenin genes. Vitellogenin knockdown leads to a decrease in the percentage of worms with HSR induction upon *F08D12.1* knockdown (*vit-3* p-value = 0.0005 and *vit-5* p-value = 0.0005) but does not affect the percentage of worms with HSR induction upon *hsp-6* knockdown (*vit-3* p-value = 0.53 and *vit-5* p-value = 0.97). Data are from at least three trials and error represents SEM.

## Discussion

**Figure 7:**
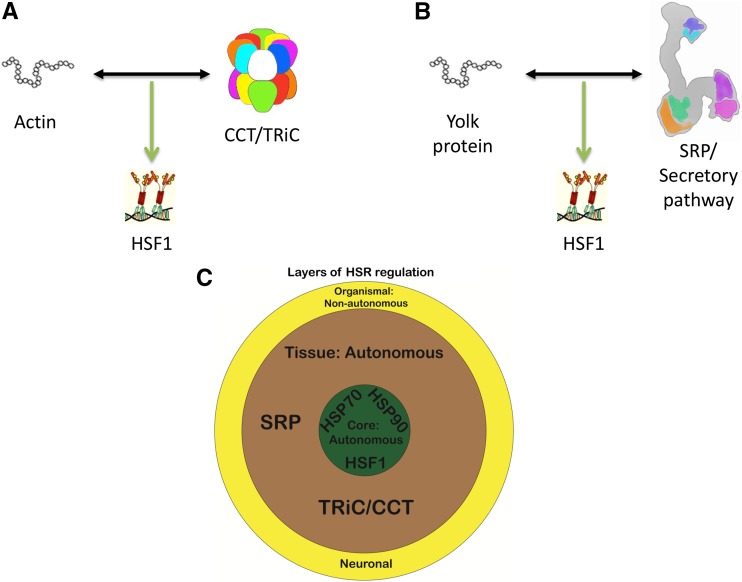
Model figure. Tissue-specific regulation represents the interplay between the proteostasis machinery (CCT/TRiC in A and SRP in B) and substrates (actin in A and yolk protein in B). (C) Interplay between organismal, tissue-specific, and core, cellular HSR regulation. HSR, heat shock response; SRP, signal recognition particle.

We have uncovered the mechanism of tissue-specific HSR regulation and found that it reflects a balance between the unique cellular proteome and the more ubiquitous proteostasis machinery. The simplest hypothesis explaining tissue-specific regulation of the HSR was tissue-specific expression of the regulators, but we found little evidence to support this hypothesis. Further, almost all HSR regulators have important functions in cellular protein synthesis, folding, trafficking, and degradation, and are therefore not likely to be expressed in a tissue-specific manner. Rather, we show that tissue-specific regulation is influenced by tissue-specific substrates. We demonstrate this principle with two orthogonal examples: an abundant TRiC/CCT substrate, actin, influences its HSR induction in the muscle and an abundant secretory protein, vitellogenin, influences HSR induction in the intestine by the secretory pathway (Figure 7). Together, these results indicate that the unique proteome of each tissue creates unique burdens upon the proteostasis network. Therefore, we predict that in the muscle, the large amount of actin creates a higher burden upon the TRiC/CCT chaperone, sensitizing this tissue to its disruption. Similarly, the large amount of vitellogenin produced and secreted in the intestine sensitizes this tissue to disruptions in the secretory pathway. Our findings parallel the well-established regulation of the HSR by the HSP70 and HSP90 chaperone machines, where HSR regulation is thought to reflect a balance between the levels of the chaperones and the amount of their substrates, indicating that regulation of the HSR by components of the proteostasis network in relation to their substrates is a general feature of HSR regulation.

Recently, the HSR has been shown to have important connections to human diseases including cancer and neurodegenerative diseases, creating a renewed interest in studying the HSR pathway. Our results are particularly relevant given the exquisite tissue-specific nature of these diseases, and our data indicate that efforts to adapt the HSR as a therapeutic approach must include an analysis of the HSR in the relevant tissues. For example, a full analysis of HSR regulation in neurons is critical to generate foundational knowledge for the successful manipulation of the HSR in neurons to combat neurodegenerative diseases. Some research in this area has already been initiated, but with mixed results. Primary neuronal cultures from mice and rats have been shown to have impaired induction of the HSR due to decreased expression of HSF1 in some cases, and impaired HSF1 activation in others (Marcuccilli *et al.* 1996, Kaarniranta *et al.* 2002, Batulan *et al.* 2003). However, similar effects have not been observed in the mouse striatum *in vivo* (Carnemolla *et al.* 2015). Unfortunately, the technical limitations of RNAi in *C. elegans* neurons have thus far precluded an analysis of HSR regulation in this tissue, but our findings motivate a new investigation of HSR regulation in *C. elegans* neurons using sensitized RNAi strains.

Previous investigations into the organismal phenotypes of actin have used knockdowns and mutants to show that the actin cytoskeleton has important roles in motility and early development (Velarde *et al.* 2007, Waterston *et al.* 1984). Here, we have undertaken complementary approaches that support these earlier observations, showing that mild actin overexpression causes defects in motility and early development. Importantly, a recent study has suggested that maintenance of the actin cytoskeleton is an important factor in maintaining lifespan (Baird *et al.* 2014). Here, we validate a prediction of that model by showing that even slight disruption of the actin cytoskeleton by mild overexpression of a single actin isoform can shorten lifespan.

Our findings on tissue-specific HSR regulation complement a growing body of literature exploring cell-autonomous and cell-nonautonomous regulation of the HSR. Cell-autonomous HSR regulation was robustly demonstrated using lasers to induce the HSR in individual cells in an intact *C. elegans* (Suzuki *et al.* 2013, Stringham and Candido 1993). We show that the mechanism of tissue-specific HSR regulation mirrors the core HSR regulation by HSP70 and HSP90, as they both act cell-autonomously to probe protein folding capacity relative to the protein folding requirements of the cell (Figure 7). This cell-autonomous regulation is quite distinct from other forms of HSR regulation at the organismal level (Prahlad *et al.* 2008, Van Oosten-Hawle *et al.* 2013, Douglas *et al.* 2015). For example, it was recently shown that mutations in neuronal genes can suppress the whole organismal response to temperature and that overexpression of HSP90 in a tissue-specific manner can affect HSR regulation in distinct tissues. These data indicate that the HSR can be coordinately regulated across tissues and at the level of the whole organism, at least under some conditions. Nonautonomous HSR regulation may be adaptive, enhancing the cellular stress response in anticipation of stress, or alternatively, it may restrict cellular responses to maximize organismal performance. As the mechanisms behind nonautonomous regulation are not yet established, the coordination between nonautonomous and autonomous HSR regulation remain largely unexplored.

Advances in molecular biology have created an explosion in our understanding of the details and mechanisms behind many signal transduction pathways. The HSR has served as a foundational pathway for understanding signal transduction since its accidental discovery >50 yr ago, due to its universal nature and the high degree of conservation among heat shock genes. Here, we use *C. elegans* to learn how the HSR is differentially adapted to each tissue in an organism and demonstrate how incorporating organismal and systems-level approaches to the study of classical signal transduction pathways can yield new insights into their functions.

## Supplementary Material

Supplemental material is available online at www.g3journal.org/lookup/suppl/doi:10.1534/g3.116.038232/-/DC1.
