# Supplementary material for: Cellular Proteomes Drive Tissue-Specific Regulation of the Heat Shock Response
Source: G3 (Bethesda). 2017 Jan 30;7(3):1011–8. doi: 10.1534/g3.116.038232 (PMC5345702; doi:10.1534/g3.116.038232)
Supplement: Supplementary file 1 [file 1011FileS1.pptx]

## Slide 1
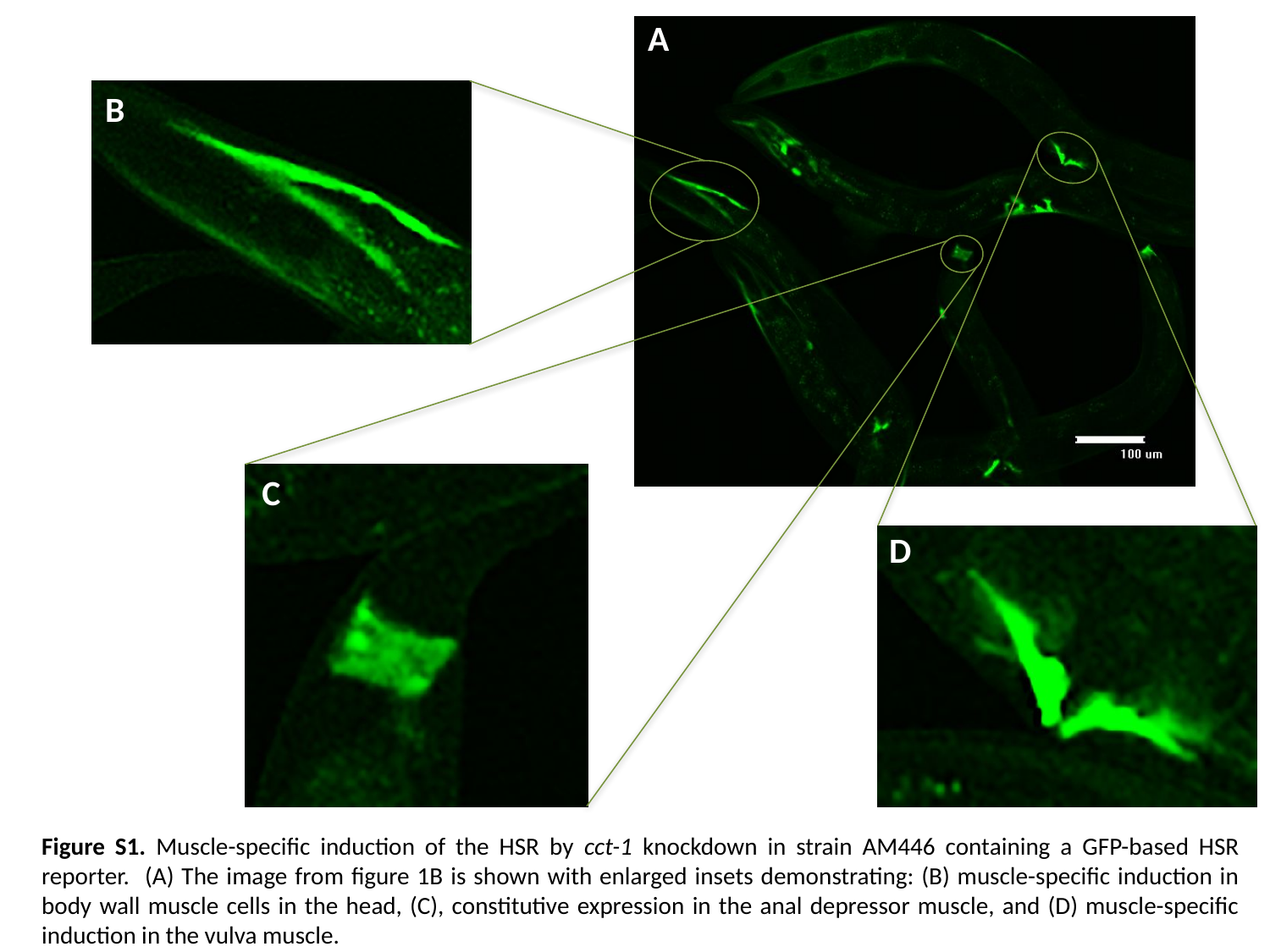

A
B
C
D
Figure S1. Muscle-specific induction of the HSR by cct-1 knockdown in strain AM446 containing a GFP-based HSR reporter. (A) The image from figure 1B is shown with enlarged insets demonstrating: (B) muscle-specific induction in body wall muscle cells in the head, (C), constitutive expression in the anal depressor muscle, and (D) muscle-specific induction in the vulva muscle.

## Slide 2
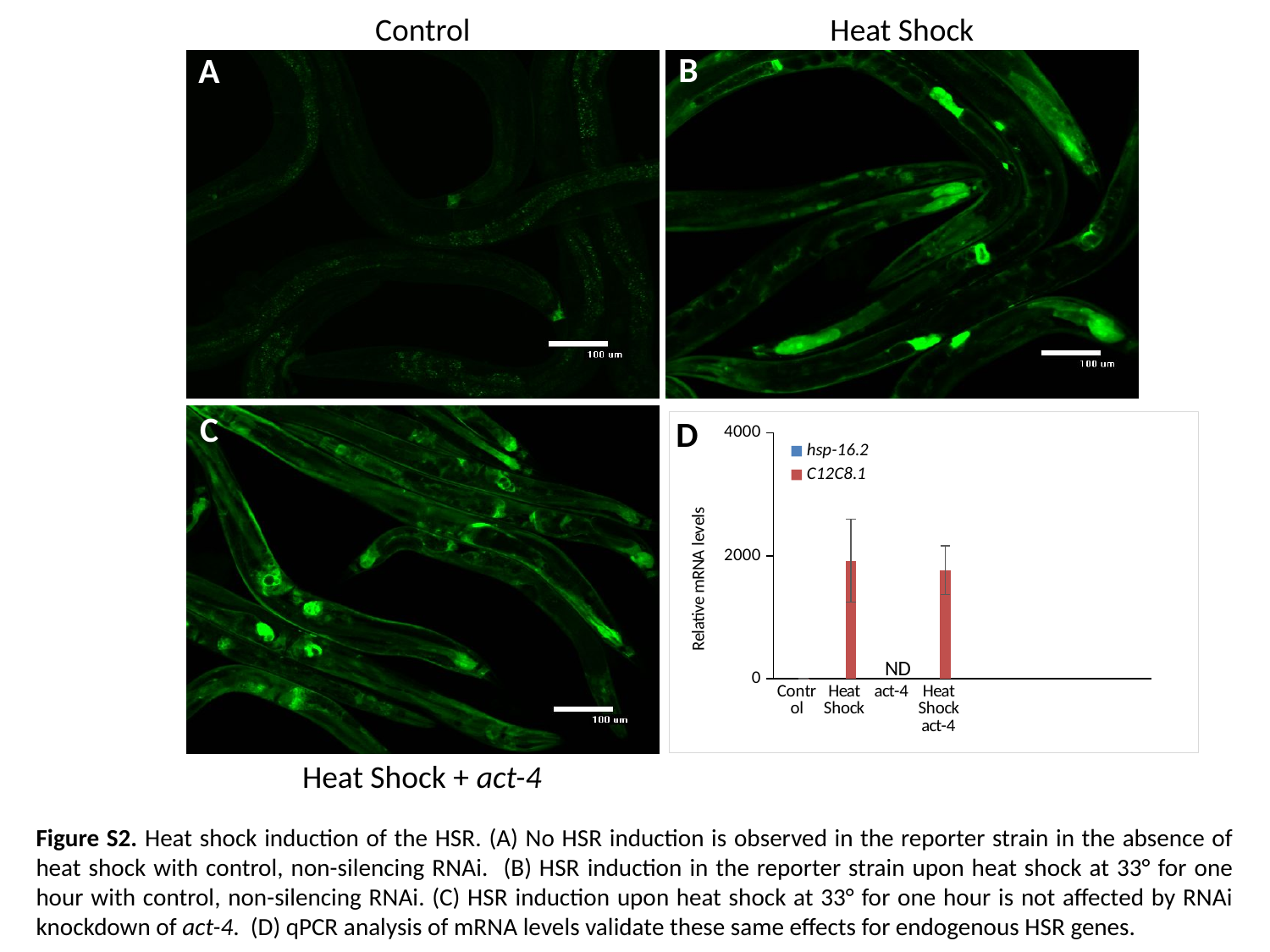

Control
Heat Shock
B
A
C
D
### Chart
| Category | hsp-16.2 | C12C8.1 |
|---|---|---|
| Control | 1.0 | 0.9999999999999999 |
| Heat Shock | 3024.2830998303193 | 1918.2293872891348 |
| act-4 | 5.126634008016385 | 0.0 |
| Heat Shock act-4 | 5346.477358544174 | 1768.2803938707725 |Heat Shock + act-4
Figure S2. Heat shock induction of the HSR. (A) No HSR induction is observed in the reporter strain in the absence of heat shock with control, non-silencing RNAi. (B) HSR induction in the reporter strain upon heat shock at 33° for one hour with control, non-silencing RNAi. (C) HSR induction upon heat shock at 33° for one hour is not affected by RNAi knockdown of act-4. (D) qPCR analysis of mRNA levels validate these same effects for endogenous HSR genes.

## Slide 3
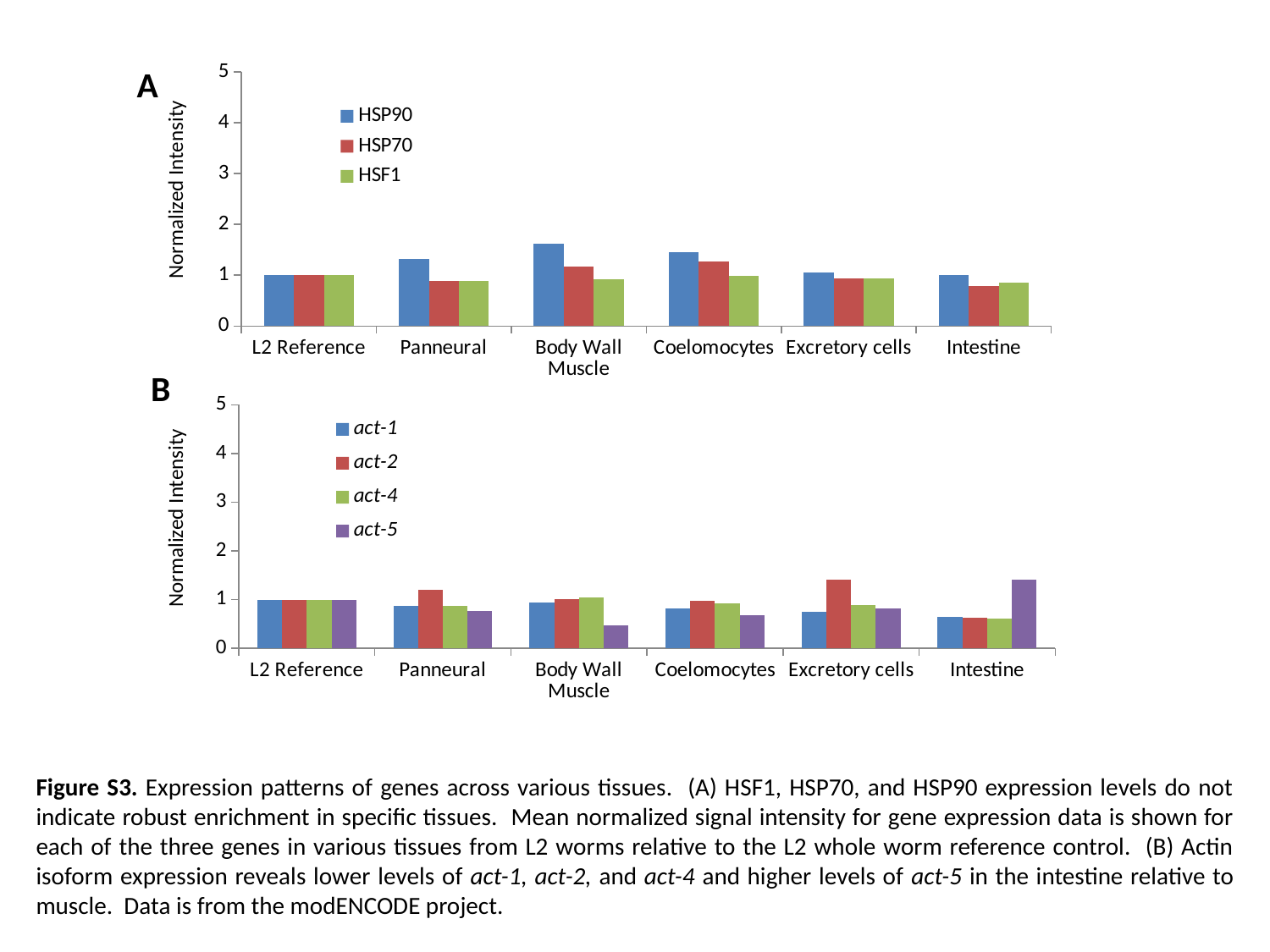

A
### Chart
| Category | HSP90 | HSP70 | HSF1 |
|---|---|---|---|
| L2 Reference | 1.0 | 1.0 | 1.0 |
| Panneural | 1.3237396883593033 | 0.8865831375619943 | 0.893152077245585 |
| Body Wall Muscle | 1.6111824014665446 | 1.1670582093448185 | 0.915131495362724 |
| Coelomocytes | 1.4482126489459213 | 1.2753850169668492 | 0.9848812095032398 |
| Excretory cells | 1.0452795600366638 | 0.9427042547637693 | 0.9302502858594841 |
| Intestine | 1.0073327222731439 | 0.794701122422344 | 0.8607546690382416 |Normalized Intensity
B
### Chart
| Category | act-1 | act-2 | act-4 | act-5 |
|---|---|---|---|---|
| L2 Reference | 1.0 | 1.0 | 1.0 | 1.0 |
| Panneural | 0.8739043023723361 | 1.208223249669749 | 0.8686420905272982 | 0.7754336913007561 |
| Body Wall Muscle | 0.9456373140329714 | 1.0148612945838837 | 1.0514465702286515 | 0.4656541907606278 |
| Coelomocytes | 0.8193003618817852 | 0.9732496697490093 | 0.9165888940737285 | 0.67897312067103 |
| Excretory cells | 0.7510253317249698 | 1.4116578599735798 | 0.8896406906206252 | 0.819088771684565 |
| Intestine | 0.6468837957378367 | 0.6284676354029062 | 0.6063929071395241 | 1.4155175700578253 |Normalized Intensity
Figure S3. Expression patterns of genes across various tissues. (A) HSF1, HSP70, and HSP90 expression levels do not indicate robust enrichment in specific tissues. Mean normalized signal intensity for gene expression data is shown for each of the three genes in various tissues from L2 worms relative to the L2 whole worm reference control. (B) Actin isoform expression reveals lower levels of act-1, act-2, and act-4 and higher levels of act-5 in the intestine relative to muscle. Data is from the modENCODE project.

## Slide 4
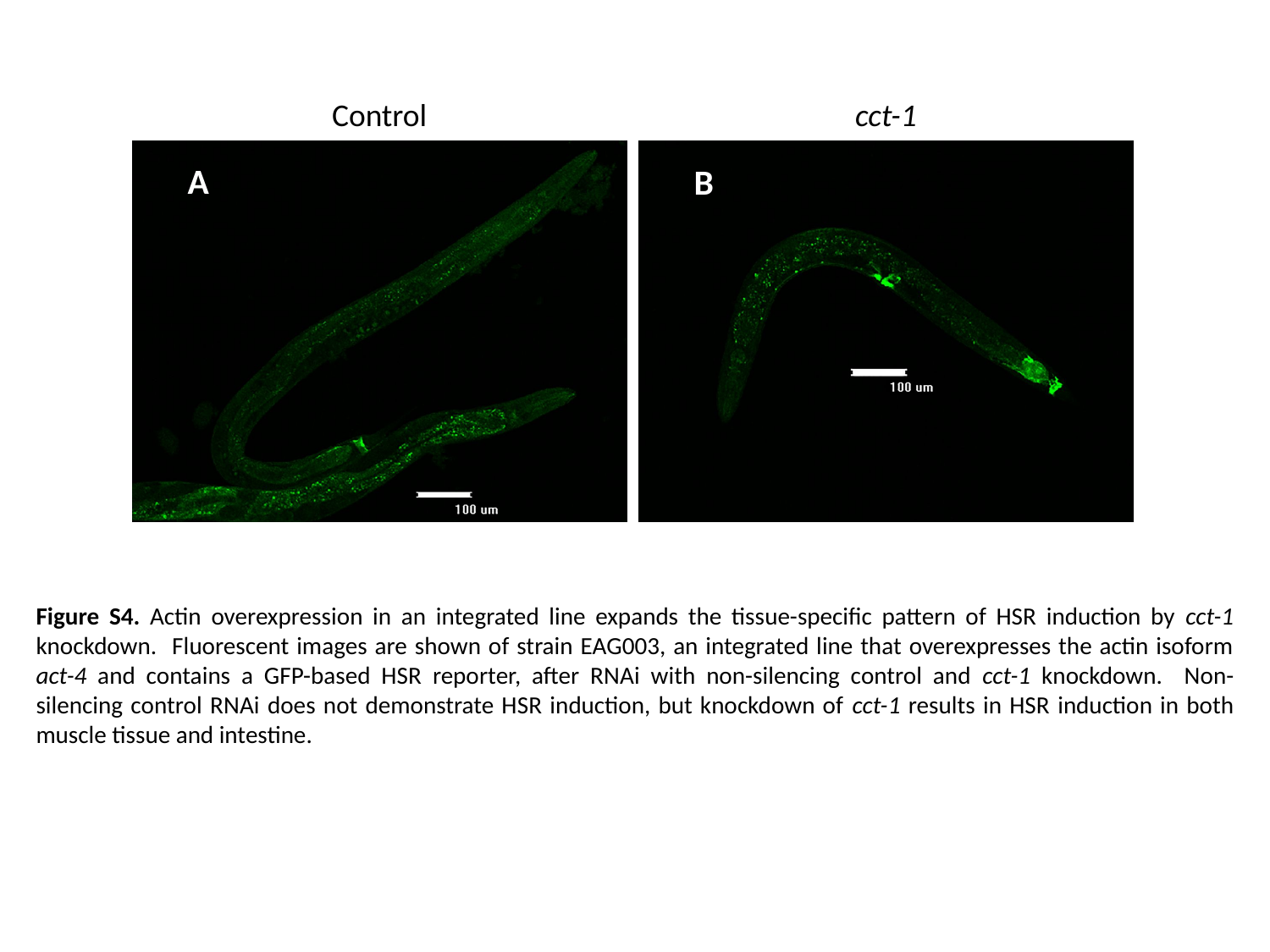

Control
cct-1
A
B
Figure S4. Actin overexpression in an integrated line expands the tissue-specific pattern of HSR induction by cct-1 knockdown. Fluorescent images are shown of strain EAG003, an integrated line that overexpresses the actin isoform act-4 and contains a GFP-based HSR reporter, after RNAi with non-silencing control and cct-1 knockdown. Non-silencing control RNAi does not demonstrate HSR induction, but knockdown of cct-1 results in HSR induction in both muscle tissue and intestine.

## Slide 5
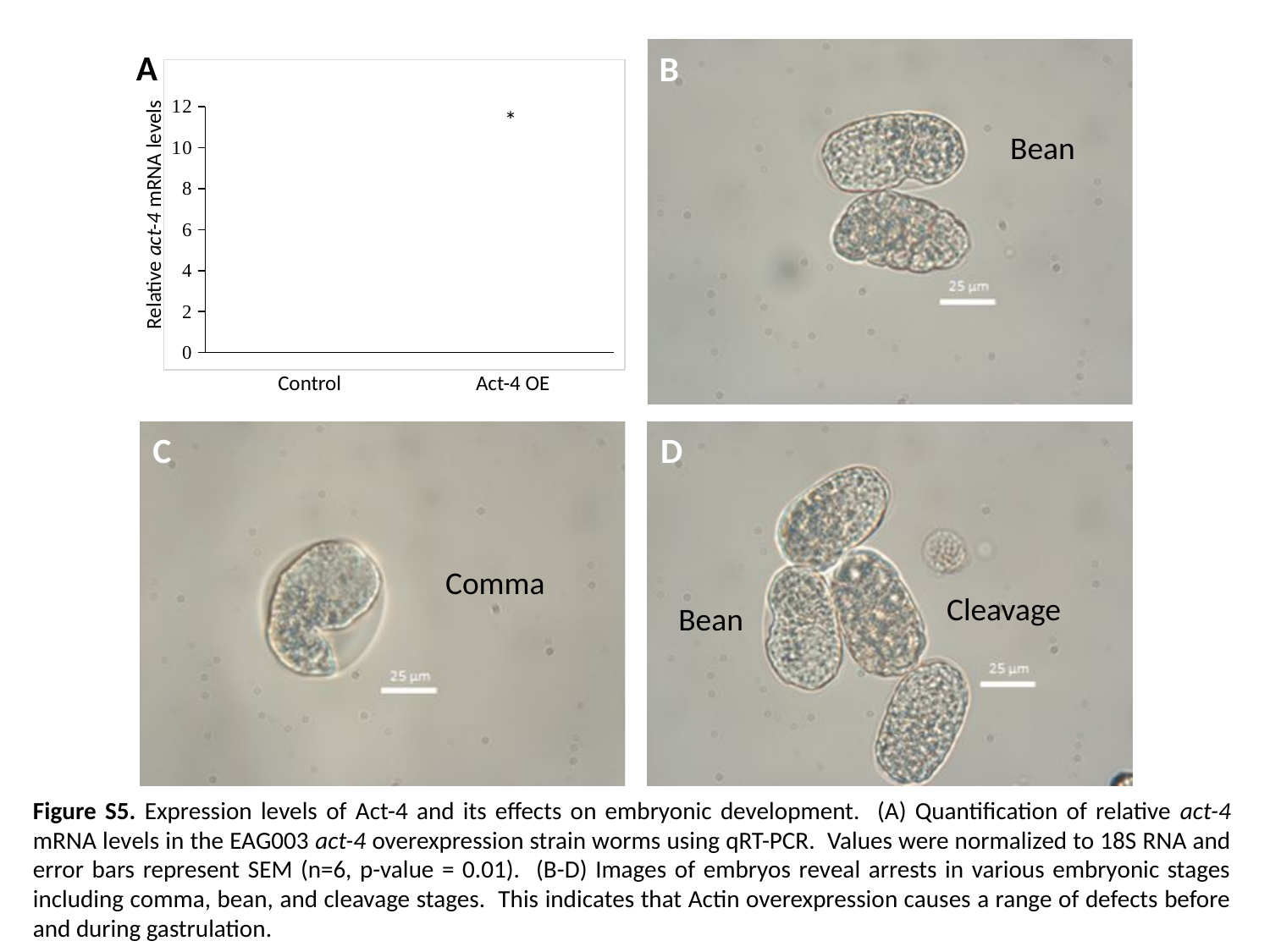

A
### Chart
| Category | Average Change |
|---|---|
| Control | 1.0 |
| Act-4 OE | 3.109266177335091 |Control
Act-4 OE
Relative act-4 mRNA levels
B
Bean
*
C
Comma
D
Cleavage
Bean
Figure S5. Expression levels of Act-4 and its effects on embryonic development. (A) Quantification of relative act-4 mRNA levels in the EAG003 act-4 overexpression strain worms using qRT-PCR. Values were normalized to 18S RNA and error bars represent SEM (n=6, p-value = 0.01). (B-D) Images of embryos reveal arrests in various embryonic stages including comma, bean, and cleavage stages. This indicates that Actin overexpression causes a range of defects before and during gastrulation.

## Slide 6
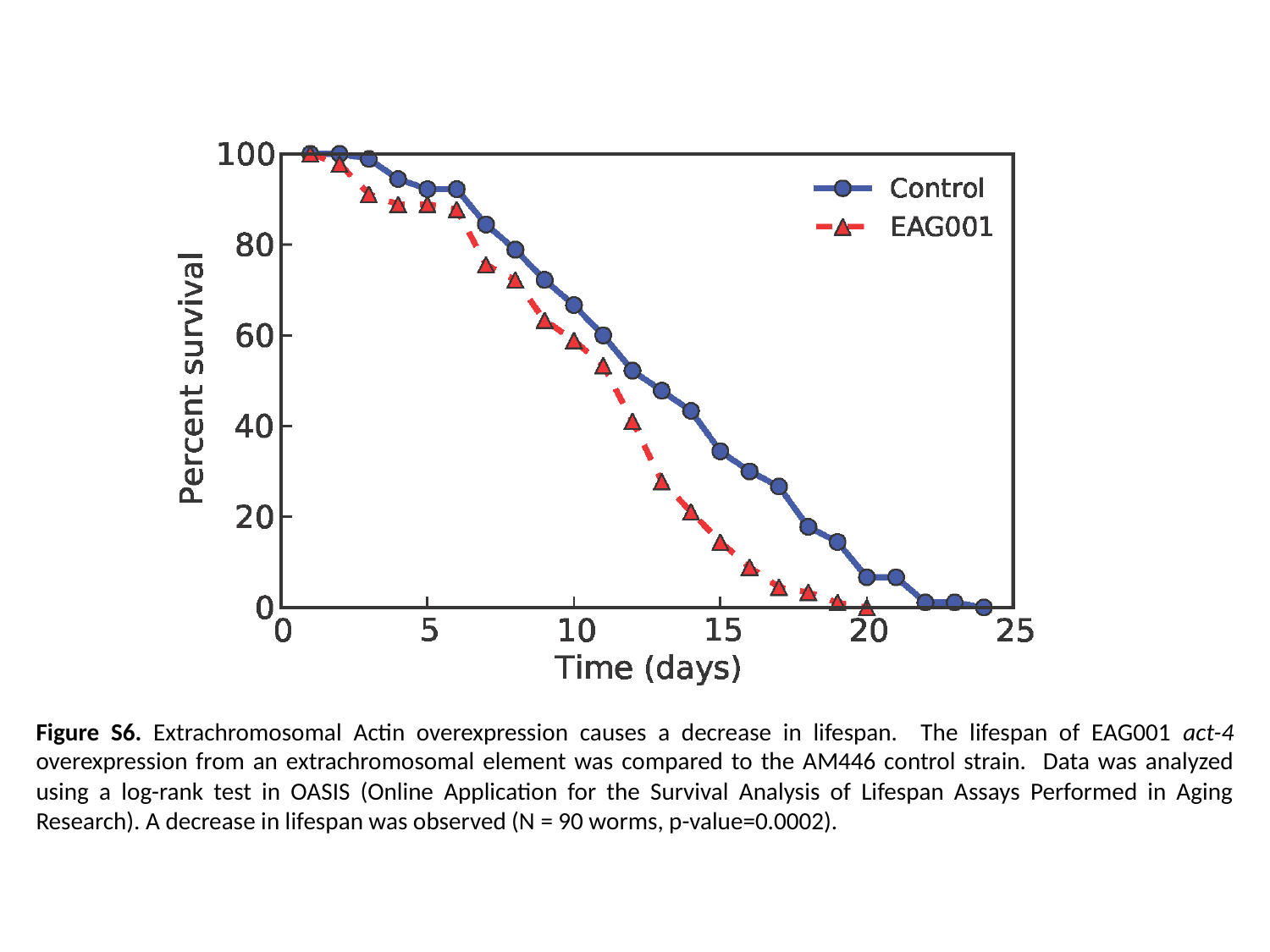

Figure S6. Extrachromosomal Actin overexpression causes a decrease in lifespan. The lifespan of EAG001 act-4 overexpression from an extrachromosomal element was compared to the AM446 control strain. Data was analyzed using a log-rank test in OASIS (Online Application for the Survival Analysis of Lifespan Assays Performed in Aging Research). A decrease in lifespan was observed (N = 90 worms, p-value=0.0002).
